# The Croatian version of the Body Image Scale: translation and validation

**DOI:** 10.3325/cmj.2021.62.598

**Published:** 2021-12

**Authors:** Iva Milić Vranješ, Matea Podgornjak, Jakov Milić, Ivona Šijan, Jelena Jakab, Ivana Krajina, Lada Zibar, Marija Heffer

**Affiliations:** 1 Department of Gynecology and Obstetrics, University Hospital Osijek, Osijek, Croatia; 2 Department of Psychiatry, General Hospital Karlovac, Karlovac, Croatia; 3 Catholic Faculty of Theology, University of Zagreb, Zagreb, Croatia; 4 Department of Radiology, National Memorial Hospital Vukovar, Vukovar, Croatia; 5 Department of Pathophysiology, Physiology and Immunology, Faculty of Dental Medicine and Health Osijek, J. J. Strossmayer University of Osijek, Osijek, Croatia; 6 Department of Dermatology and Venereology, Osijek University Hospital, Osijek, Croatia; 7 Department of Nephrology, University Hospital Merkur, Zagreb, Croatia; 8 Department of Medical Biology and Genetics, Faculty of Medicine Osijek, J. J. Strossmayer University of Osijek, Osijek, Croatia

## Abstract

**Aim:**

To validate the Croatian translation of the Body Image Scale in breast cancer and chronic kidney disease patients.

**Methods:**

The scale was administered to 172 breast cancer patients and to 89 chronic kidney disease patients. Measures of depression and anxiety were used to assess the convergent validity. Both groups were divided based on their treatment types.

**Results:**

In both samples, the scale showed high internal consistency (Cronbach's Alpha 0.958 for breast cancer patients, 0.855 for chronic kidney disease patients) item-total correlations (0.72-0.88 for breast cancer patients, 0.46-0.65 for chronic kidney disease patients), and convergent validity. In the breast cancer group, the factor analysis showed a single-factor solution, while in the chronic kidney disease group it showed a two-factor solution. Good discriminant validity was obtained among breast cancer patients, with patients who underwent complete mastectomy scoring higher than patients who underwent partial mastectomy. The scale showed no discriminant validity among chronic kidney disease patients.

**Conclusion:**

The Croatian BIS shows good psychometric properties.

Body image (BI) is a multidimensional construct consisting of perceptions, feelings, and thoughts related to the general appearance, function, and physical competence of one's body (1,2). Changes in body appearance and functioning as a result of cancer treatment and surgery can negatively affect patients' BI. In patients undergoing conserving surgery and mastectomy, BI-related problems are often underestimated as survival is put to the forefront. Unfavorable physical changes in these patients greatly affect their self-perception (3-6). Negative BI in cancer patients correlates with a lower quality of life (QOL) (7). It can also prevent posttraumatic growth, which is defined as a positive change resulting from stressful and traumatic events (6,8).

BI is assessed either by items in global measures of QOL or by separate BI questionnaires (9). Despite the importance of BI in cancer patients, relatively few BI assessment tools have been thoroughly validated for application in oncology. Several tools are specifically designed for use in breast cancer patients: the Body Image After Breast Cancer Questionnaire, the Body Image and Relationships Scale, the Breast-Impact of Treatment Scale, and Sexual Adjustment and Body Image Scale (SABIS). The SABIS was initially developed for breast cancer patients, but there is also a gynecologic version, SABIS-G (5). The assessment tools that have been validated in several types of cancer patients include Body Image Screener for Cancer Reconstruction and the Measure of Body Apperception, and the Body Image Scale (BIS) (10,11).

The BIS is a 10-item scale developed by Hopwood et al (12) that measures affective, behavioral, and cognitive aspects of BI in all cancer patients, applicable in research and clinical settings. The respondents rate the severity of their symptoms on a four-point scale, from 0 (“not at all”) to 3 (“very much”), and the 10 item scores are combined into a total score ranging from 0 to 30 (12).

The questionnaire was initially validated in a British sample of heterogeneous cancer patients and breast cancer patients, showing good measurement properties (12). Since then, the BIS has been validated in several languages, including Dutch, Greek, and Portuguese (11,12). Furthermore, it was applied in more cross-cultural studies compared with other BI assessment tools used in oncology (10). Although BIS has been designed for BI assessment in cancer patients, it has also been used in other populations, eg, to assess psychosocial outcomes in kidney donors after surgery (13).

The aim of this study was to validate the Croatian translation of BIS in breast cancer patients and chronic kidney disease (CKD) patients. To the best of our knowledge, this is the first Croatian translation of the BIS, an important fact when considering the relatively few validated tools for BI assessment designed specifically for these patients. Furthermore, this valuable instrument can be used for further research in this largely under-researched scientific area in the Croatian population. A complete Croatian version is available in the supplement and can be freely used in other research.

## Patients and methods

Although BIS was designed to assess BI in oncological patients, we noticed that the questions did not refer explicitly to cancer, but only to the scar. We hypothesized that BIS should be applicable for BI evaluation in non-cancer patients who underwent some kind of surgery. Since renal transplantation in CKD patients leaves a significant scar and since women treated with hemodialysis all underwent surgery, at least in order to gain vascular access in the form of an arteriovenous fistula, we believed that BIS could be used in these patients. Women treated with hemodialysis might also have other physical consequences of uremia, such as dry skin or paleness, which could also influence their BI.

Therefore, for this validation study, we collected data from two separate studies using the same Croatian version of the BIS. Both parts of the study were approved by the Ethics Committees of the Faculty of Medicine Osijek and Osijek University Hospital. The study protocol conformed to the provisions of the Declaration of Helsinki. All participants gave written informed consent.

### Translation

The questionnaire was translated in several steps. First, three fluently bilingual Croatian medical professionals translated the instrument into Croatian. The three translated versions were discussed by the team, and a combined version was created. A native Croatian translator with a master’s degree in English back-translated the scale into English. The original version and the back-translation were compared, and the translation was amended to ensure clarity. The algorithm was decided upon after considering procedures suggested in several articles (14-21).

The BIS questionnaire is a patient-reported outcome measure designed to determine BI in cancer patients (12). The questionnaire was developed in collaboration with the European Organization for Research and Treatment of Cancer, and is adapted for use in patients with all types of cancer (9,12). BIS contains 10 questions with answers on a four-point scale (0 = Not at all; 1 = A little; 2 = Quite a bit; 3 = Very much). The respondents were instructed to circle the number that reflected the extent to which they were critical of their appearance, dissatisfied with the look of their body and scar, felt less physically attractive, and felt less whole as a consequence of their illness and treatment. The final result is obtained by adding up the scores awarded to individual questions, with a minimum score of 0 and a maximum score of 30 points. This brief questionnaire comprehensively assesses the affective, behavioral, and cognitive dimensions of self-image (9).

### Breast cancer patients

A cross-sectional study involved 172 women surgically treated for breast cancer in the Institute for Thoracic, Plastic, and Reconstructive Surgery, Osijek University Hospital. The participants were divided into the mastectomy group (n = 88) and breast conserving surgery group (n = 84). The exclusion criteria were metastatic disease, terminal stage of the disease, age under 18 and over 85, other serious somatic diseases, mental illness, and lack of informed consent.

The participants were administered a questionnaire consisting of the BIS and the Depression Anxiety Stress Scale (DASS-21). The DASS-21 is a short, 21-item, version of the validated DASS questionnaire, evaluating the levels of depression, anxiety, and stress (22). The level of each component is determined by seven statements. The respondents circle a number on a four-point scale (0 = Did not apply to me at all; 1 = Applied to me to some degree, or some of the time; 2 = Applied to me to a considerable degree or a good part of time; 3 = Applied to me very much or most of the time), indicating the extent to which the statement applied to them over the previous week. The total score for each component is determined by adding up the points awarded to individual statements for that component, with a minimum score of 0 and a maximum of 21 (22-24).

### Patients with chronic kidney disease

A cross-sectional study involved 68 female patients with CKD treated at the Department of Nephrology of the Internal Clinic, Osijek University Hospital. Thirty-five patients underwent chronic hemodialysis and 33 underwent kidney transplantation surgery. The exclusion criteria were malignant disease, age under 18 years, previous mutilating surgical procedures, other severe somatic diseases or psychiatric illnesses, and lack of informed consent.

The participants completed a self-administered questionnaire consisting of BIS, Patient Health Questionnaire (PHQ-9), and Generalized Anxiety Disorder (GAD-7) instruments.

The PHQ-9 is a validated instrument with a high sensitivity and specificity for depression screening. It consists of nine questions with answers on a four-point scale (0 = Not at all, 1 = Several days, 2 = More than half the days, 3 = Nearly every day), each providing information about one criterion for depression defined by DSM-IV (25-28). The cut-off score for depression was 10 or above (25,26,29).

The GAD-7 is a validated questionnaire developed to briefly evaluate the symptoms of anxiety occurring during the two weeks before completion and is used as a screening for generalized anxiety disorder. The questionnaire consists of seven questions with answers on a four-point scale (0 = Not at all, 1 = Several days, 2 = More than half the days, 3 = Nearly every day) (30-33). The respondent circles the number next to a statement that best reflects the extent to which she felt nervous or worried too much, had trouble relaxing, became easily annoyed, and felt afraid that something awful might happen (34).

Alongside BIS, other questionnaires for the evaluation of psychological distress (ie, DASS-21, GAD-7, PHQ-9) were also used as external validation points, since greater BI disturbance was associated with increased psychological discomfort (12,35-37).

### Statistical analysis

The normality of distribution was tested with the Kolmogorov-Smirnov test. Two or more independent groups were compared with the Mann-Whitney. Correlation between variables was assessed with the Spearman’s rank-order correlation coefficient. Confirmatory factor analysis (CFA) was used to test the fit of the one-factor model of the BIS. Different aspects of fit were evaluated, including absolute fit (χ^2^), fit adjusted for model parsimony (Tucker-Lewis Index or TLI), fit relative to a null model (comparative fit index or CFI), and root mean square error of approximation (RMSEA). The criteria for adequate fit were CFI and TLI values of more than 0.90 and a RMSEA less than 0.08 (38,39). Because the BIS scores were at the interval level, the maximum-likelihood (ML) was used as the estimator. To test the sampling adequacy, the Kaiser-Meyer-Olkin (KMO) test was used. The Bartlett’s test was used to assess redundancy. *P* < 0.05 was considered statistically significant. The analysis was conducted with SPSS, version. 16.0 (SPSS Inc., Chicago, IL, USA). CFA was performed with AMOS, version 18.0 (SPSS Inc., Chicago, IL, USA).

## Results

### Breast cancer patients

The mean age of breast cancer patients was 47.1 ± 8.783 years. The median scores and interquartile ranges (IQR) of the DASS domains were as follows: Depression 8 (2-18), Anxiety 8 (2-18), and Stress 14 (8-20). The median score and IQR of the BIS were 9 (2-13). Table 1 presents the correlations between the mentioned variables.

**Table 1:** Correlations (Spearman's rho) of age, Body Image Scale, and the domains of the Depression Anxiety Stress Scale (DASS-21) in breast cancer patients (N = 172)

	Body Image Scale	Depression	Anxiety	Stress
Age	-0.243*	-0.096	-0.166^†^	-0.128
Body Image Scale		0.598*	0.458*	0.557*
Depression			0.690*	0.858*
Anxiety				0.756*

*P < 0.01, Spearman's rho.

†*P* < 0.05, Spearman's rho.

The median time since surgery was 28 months (13.25-51.75). The BIS score did not significantly correlate with the time since surgery (*P* = 0.203, Spearman's rho = -0.98).

To test the discriminant validity of the scale, we compared the patients based on the type of their operation. Patients with a complete mastectomy had significantly higher scores than patients with partial mastectomy (Mann-Whitney U 2402.5, *P* < 0.001, η^2^ = 0.072), which indicates lower satisfaction with their BI (11 [IQR 4-19.5]) vs 6 [IQR 1-11]).

**Figure 1:**
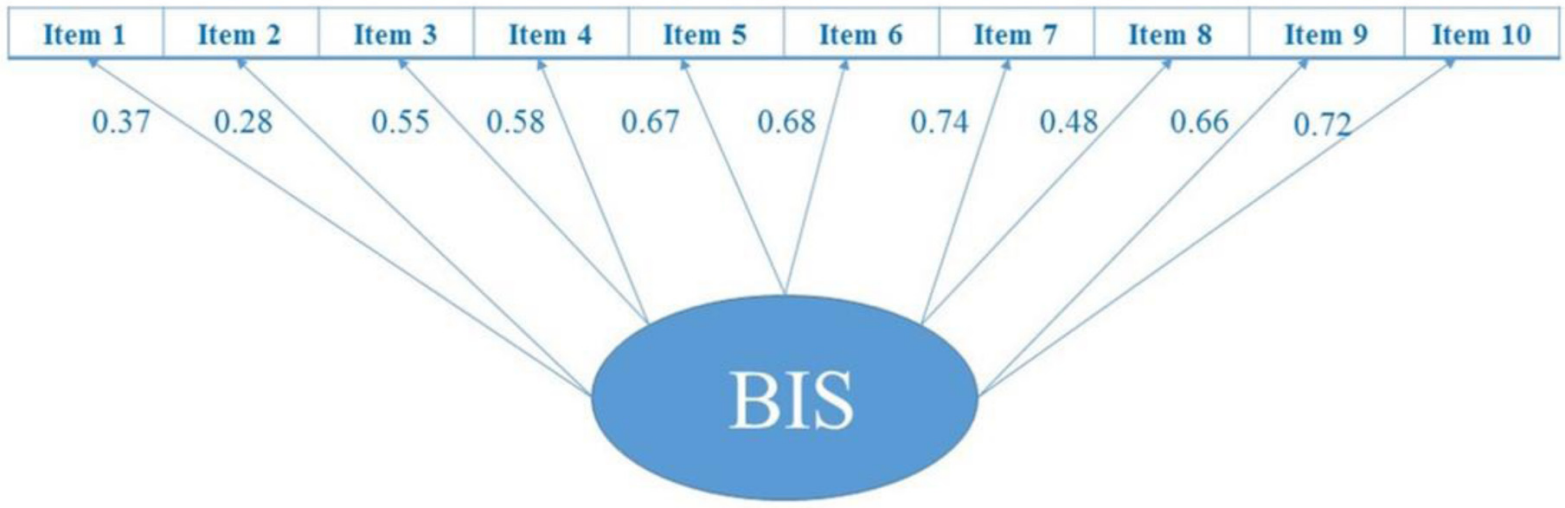
The fit indices of the one-factor model for the Body Image Scale in patients with mastectomy

The fit indices of the one-factor model suggested a satisfactory fit for the data for patients with mastectomy, χ^2^(35, N = 172) = 153.50, *P* < 0.001, TLI = 0.91, CFI = 0.93, RMSEA = 0.068 (Figure 1).

The Croatian version of the BIS in breast cancer patients showed high internal consistency (Cronbach's Alpha = 0.958). Item statistics for both studies are presented in Table 2. In breast cancer patients, corrected item-total correlations ranged from 0.72 to 0.88.

**Table 2:** Descriptive and item statistics pertaining to the items of the translated Body Image Scale for breast cancer (BC) and chronic kidney disease (CKD) patients

Item	Mean ± standard deviation	Scores greater than zero (%)	Corrected item-total correlations	Alpha if item deleted
1. Have you been feeling self-conscious about your appearance?				
BC*	1.15 ± 0.89	77.3	0.74	0.96
CKD*	0.81 ± 0.83	57.4	0.55	0.84
2. Have you felt less physically attractive as a result of your disease or treatment?				
BC	1.11 ± 0.99	67.4	0.87	0.95
CKD	0.69 ± 0.99	41.2	0.50	0.85
3. Have you been dissatisfied with your appearance when dressed?				
BC	0.82 ± 0.93	55.8	0.80	0.95
CKD	0.81 ± 0.85	57.4	0.64	0.84
4. Have you been feeling less feminine/masculine as a result of your disease or treatment?				
BC	1.02 ± 0.99	64.0	0.88	0.95
CKD	0.74 ± 0.92	47.1	0.63	0.84
5. Did you find it difficult to look at yourself naked?				
BC	0.87 ± 1.03	51.7	0.80	0.95
CKD	0.82 ± 0.99	50.0	0.65	0.84
6. Have you been feeling less sexually attractive as a result of your disease or treatment?				
BC	1.16 ± 1.06	66.3	0.88	0.95
CKD	0.84 ± 1.70	39.7	0.50	0.86
7. Did you avoid people because of the way you felt about your appearance?				
BC	0.45 ± 0.79	32.6	0.72	0.96
CKD	0.47 ± 0.95	26.5	0.60	0.84
8. Have you been feeling the treatment has left your body less whole?				
BC	0.92 ± 0.98	58.1	0.87	0.95
CKD	0.88 ± 1.13	48.5	0.63	0.84
9. Have you felt dissatisfied with your body?				
BC	0.99 ± 0.97	65.1	0.87	0.95
CKD	0.84 ± 1.10	51.5	0.64	0.84
10. Have you been dissatisfied with the appearance of your scar?				
BC	0.81 ± 0.94	53.5	0.72	0.96
CKD	0.57 ± 0.98	35.3	0.46	0.85

### Chronic kidney disease patients

The mean age of CKD patients was 59.62 ± 13.663 years. The median (IQRs) scores were as follows: BIS 5.5 (2-10), GAD7 5.5 (3-8), PHQ 9.8 (4-12).

To test the discriminant validity of the scale, we compared the patients based on the type of treatment. No significant difference was observed between the hemodialysis and transplantation group (7 [4-13] vs 3 [1-9], respectively, Mann-Whitney U 429.5, *P* = 0.069, η^2^ = 0.048). The correlations between the tested variables are presented in Table 3.

**Table 3:** Correlations (Spearman's rho) of age, the Body Image Scale (BIS), General Anxiety Disorder-7 (GAD-7), and Patient Health Questionnaire-9 (PHQ-9) scores in chronic kidney disease patients (N = 68)

	BIS	GAD7	PHQ9
Age	-0.061	0.265^†^	0.03
BIS		0.462^†^	0.445*
GAD7			0.579*

^*^^P^ ^< 0.01, Spearman's rho.^

†*^P^* ^< 0.05, Spearman's rho.^

**Figure 2:**
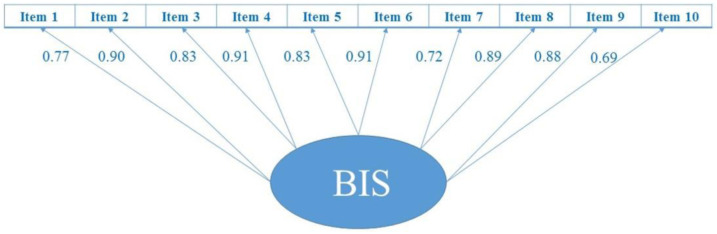
The fit indices of the one-factor model for the Body Image Scale in patients with chronic kidney disease

In contrast to patients with mastectomy, the fit indices of the one-factor model for chronic kidney disease patients showed poor fit, χ^2^(35, N = 68) = 67.24, *P* <  0.001, TLI = 0.78, CFI = 0.84, RMSEA = 0.117 (Figure 2). Given this, exploratory factor analyses was conducted.

The KMO test showed good sampling adequacy, although it was still lower than in breast cancer patients (0.738). Bartlett’s test showed no redundancy (*P* < 0.001). Contrary to the results of the first study, PCA showed a two-factor solution. The first factor had an Eigeinvalue of 4.621, which explained 46.213% of the variance. The highest loading was observed for question 9 (0.774), while the lowest was observed for question 10 (0.493, Table 4). The second factor had an Eigenvalue of 1.414 and it explained 14.141% of the variance. In the second sample, corrected item-total correlations ranged from 0.46 to 0.65. The internal consistency was adequate (Cronbach's Alpha = 0.855).

**Table 4:** Factor analysis of the Croatian Body Image Scale in breast cancer patients (N = 172) and chronic kidney disease patients (N = 68)

	Factor (eigenvalue)		
	Breast cancer patients	Chronic kidney disease patients	
Item number	1 (7.281)	1 (4.621)	2 (1.41)
1	0.787	0.644	0.154
2	0.901	0.614	0.053
3	0.844	0.729	0.167
4	0.904	0.720	0.031
5	0.842	0.752	-0.165
6	0.909	0.572	0.632
7	0.766	0.698	-0.337
8	0.897	0.747	-0.391
9	0.895	0.774	-0.427
10	0.770	0.493	0.696
% of variance	72.81	46.2	14.14

In CKD patients, we observed two factors. Upon further inspection, only items 6 and 10 failed to load on factor 1. Since three items are normally required to load on a factor, we cannot conclude that our study shows two factors, but that the scale is not suitable for this population.

## Discussion

The Croatian version of the BIS showed high internal consistency in breast cancer patients, with the Cronbach’s Alpha of 0.958 for the entire sample. This value was higher than in the original development of the scale, and comparable to other translations of the BIS (12,40,41). Item-total correlations were also comparable to other validation studies (40,42). As test-retest reliability could not be examined, it needs to be assessed in future studies. Factor analysis showed a single-factor solution, explaining 72.81% of the variance. This is a higher value than observed in the original study and higher than or comparable to other validation studies (40,41). Even though most studies found a single-factor solution, the Greek version had a two-factor solution (11,42).

The Croatian translation of the BIS also showed convergent validity in breast cancer patients, with BIS scores strongly positively correlating with the scores on the DASS domains. This is in line with the results of a previous study showing the correlation of negative BI with psychological distress (41,42). However, in this study the BIS was not compared with other BI measures. This issue needs to be addressed in future studies to further confirm the convergent validity of the Croatian version of the BIS. The Croatian BIS also showed good discriminant validity among breast cancer patients, with patients who underwent complete mastectomy scoring higher than patients who underwent partial mastectomy. This is in line with the results of the original study, as well as with those of other validation studies (9,12,42).

Although the BIS was originally developed for cancer patients, by analyzing the questions, we assumed it could be useful in studying the BI of CKD patients. In these patients, the scale showed adequate internal consistency (Cronbach’s Alpha = 0.855) and item-total correlations (0.460 to 0.647). However, contrary to the results in breast cancer patients, the factor analysis showed a two-factor solution, the first factor explaining 46.213% of the variance and the second factor explaining 14.141% of the variance. We hypothesized that the translation would be applicable to this group of patients because of the presence of post-surgical scars and uremia-related body changes. This hypothesis, however, was not confirmed, probably owing to the small sample size. Although the number of patients was sufficient for analysis, since on average 5 respondents per item are required, due to the relatively high participants' age some items might have been misunderstood. The scars resulting from gaining vascular access for dialysis or from renal transplantation surgery are possibly not disfiguring enough to affect BIS results. Consequently, these patients might not have considered the question 10 to be important, since it specifically referred to the dissatisfaction with the scar's appearance. However, it is unclear why question 6 did not fit into the factor. This could be explained by the small sample size.

The BIS scores also positively correlated with GAD7 and PHQ-9 scores, showing convergent validity of the scale. On the other hand, BIS scores did not differ based on the type of treatment, showing lack of discriminant validity. CKD patients had lower BIS response prevalence scores (with item 7 below the threshold of 30%) and a lower range of scores than breast cancer patients. The scores and the response prevalence are comparable to other studies in which the BIS was administered to non-cancer patients (43,44). However, contrary to our study, the factor analysis in a study on inflammatory bowel disease patients showed a single-factor solution, explaining 65% of the variance (43).

In conclusion, the Croatian translation of the BIS showed good psychometric properties in breast cancer patients, but further studies are needed to assess the temporal stability of the scale. The translation also showed reliability among CKD patients, with results comparable to other studies performed in non-cancer patients. Although this translation is a valid tool to measure BIS in breast cancer patients, it failed to show good validity in CKD patients. Due to the results of the factor analysis and a lack of discriminant validity, larger studies need to confirm the clinical validity of the Croatian version of the BIS in CKD patients.
